# Altered Hypothalamic Functional Connectivity with Autonomic Circuits and the Locus Coeruleus in Migraine

**DOI:** 10.1371/journal.pone.0095508

**Published:** 2014-04-17

**Authors:** Eric A. Moulton, Lino Becerra, Adriana Johnson, Rami Burstein, David Borsook

**Affiliations:** 1 Pain/Analgesia Imaging Neuroscience (P.A.I.N.) Group, Department of Anesthesia, Boston Children’s Hospital, Center for Pain and the Brain, Harvard Medical School, Waltham, Massachusetts, United States of America; 2 Athinoula A. Martinos Center for Biomedical Imaging, Massachusetts General Hospital, Charlestown, Massachusetts, United States of America; 3 P.A.I.N. Group, Department of Psychiatry, McLean Hospital, Center for Pain and the Brain, Harvard Medical School, Belmont, Massachusetts, United States of America; 4 Anaesthesia & Critical Care, Beth Israel Deaconess Medical Center, Harvard Medical School, Boston, Massachusetts, United States of America; Universiteit Gent, Belgium

## Abstract

The hypothalamus has been implicated in migraine based on the manifestation of autonomic symptoms with the disease, as well as neuroimaging evidence of hypothalamic activation during attacks. Our objective was to determine functional connectivity (FC) changes between the hypothalamus and the rest of the brain in migraine patients vs. control subjects. This study uses fMRI (functional magnetic resonance imaging) to acquire resting state scans in 12 interictal migraine patients and 12 healthy matched controls. Hypothalamic connectivity seeds were anatomically defined based on high-resolution structural scans, and FC was assessed in the resting state scans. Migraine patients had increased hypothalamic FC with a number of brain regions involved in regulation of autonomic functions, including the locus coeruleus, caudate, parahippocampal gyrus, cerebellum, and the temporal pole. Stronger functional connections between the hypothalamus and brain areas that regulate sympathetic and parasympathetic functions may explain some of the hypothalamic-mediated autonomic symptoms that accompany or precede migraine attacks.

## Introduction

Migraine, a common neurological disorder, is characterized by episodic headache attacks, and is frequently accompanied by nausea, vomiting, hunger, yawning, thirst, photophobia, phonophobia, and/or sleep disorders [1], [2]. In addition, conjunctival injection, lacrimation, nasal congestion, rhinorrhoea, eyelid edema and forehead/facial sweating are also common in migraineurs [3]. These symptoms implicate alterations in brain autonomic systems.

By regulating many sympathetic and parasympathetic responses, the hypothalamus is thought to heavily involved in physiological functions such as food ingestion, energy balance, stress, circadian rhythms, arousal, and autonomic responses to pain. The central role of the hypothalamus in regulating autonomic functions and homeostasis suggests that it may underlie some autonomic symptoms associated with migraine [2], [4]–[7] or its prodromal phase [4], [5], [7], [8]. Evidence linking the hypothalamus to migraine include (a) imaging data showing that the hypothalamus is activated during spontaneous migraine without aura [9], (b) prevalence of obesity among chronic migraineures [5], [6], [10], (c) the cyclic nature of the condition [11], and (d) its greater prevalence in women after puberty and in homosexual men [12]–[14]. Partially responsible for changes in hypothalamic functions may be attributed to the large input it receives from ascending trigeminovascular neurons in the spinal trigeminal nucleus [7]. The cyclic nature of the disease relates to how repetitive processes including hormonal cycle in women or the sleep-wake cycle [15] may alter allostatic load in the disease [16].

While the hypothalamus appears to be an important structure in migraine, imaging studies have yet to explicitly evaluate whether the hypothalamus has altered functional processing during the interictal state. One approach is to evaluate changes in functional connectivity of this structure in patients compared with healthy controls. In fMRI, functional connectivity (FC) is defined as temporal correlations between spatially remote neurophysiological events or functional interactions [17]. Given that the hypothalamus may be significantly involved in migraine attacks, we hypothesized that fMRI FC between the hypothalamus and autonomic processing areas in the brain are enhanced in interictal migraine patients as compared with healthy control subjects. As such, the alteration in FC would not only reflect the effects of repeated activation in the migraine attack, but potentially represent a sensitization of the functional connections between the hypothalamus and other brain structures involved in autonomic function.

## Materials and Methods

Using fMRI, we recorded blood oxygen level dependent (BOLD) signal fluctuations during resting state in 12 episodic migraine patients and 12 healthy age- and gender-matched control subjects.

### Ethics Statement

This study was approved by the McLean Hospital Institutional Review Board, and met the scientific and ethical guidelines for human research of the Helsinki Accord (http://ohsr.od.nih.gov/guidelines/helsinki.html). All patients and subjects provided written informed consent to participate in this study.

### Subjects

Episodic migraine patients (9 females, 3 males; 31·7±7·6 years old; **Table 1**) were free of neurological and other sensory dysfunctions. The patients included in the study had acute intermittent migraine without aura as defined by the International Headache Society (<14 attacks/month). Subjects were not having a migraine attack at least 72 hours prior to testing. In addition no patient had a migraine precipitated during or on the day following the baseline scan.

**Table 1:** Subject demographics.

	Age	Sex	Freq	Onset	Side	Pain w/o med	Pain w/med	Medications
**Patients**								
M1	31.9	M	1/mo	15 yrs	B	9	4	Acetaminophen, Ibuprofen
M2	49	F	5/mo	39 yrs	U	10	0	Sumatriptan, Lisinopril
M3	36.3	F	1/mo	33 yrs	L	10	9	Aspirin, Acetaminophen, Ibuprofen
M4	24.8	F	7–8/mo	8 yrs	B	8	4	Amitriptyline, Atenolol, Acetaminophen, Naproxen, Rizatriptan
M5	22.9	F	3–4/mo	7 yrs	B	10	7	Acetaminophen, Ibuprofen
M6	25.7	F	3–4/mo	7 yrs	B	10	10	Ibuprofen
M7	32.1	F	2–4/mo	21 yrs	U	7	6	None
M8	37.6	M	2/mo	32 yrs	R	10	9	None
M9	24.6	F	1/mo	4 yrs	L	10	6	Acetaminophen
M10	26.8	M	5/mo	3 yrs	B	5–6	N/A	None
M11	38.8	F	2/mo	28 yrs	U	9–10	4	Ibuprofen, Midrin
M12	30.2	F	1–3/mo	8 yrs	R	7	7	Rizatriptan
**Healthy Controls**								
H1	32.2	M	–	–	–	–	–	–
H2	27.9	F	–	–	–	–	–	–
H3	24.5	F	–	–	–	–	–	–
H4	23.3	F	–	–	–	–	–	–
H5	36.9	F	–	–	–	–	–	–
H6	26.4	F	–	–	–	–	–	–
H7	38	M	–	–	–	–	–	–
H8	30.8	F	–	–	–	–	–	–
H9	24.3	M	–	–	–	–	–	–
H10	31.6	F	–	–	–	–	–	–
H11	36.3	M	–	–	–	–	–	–
H12	49	F	–	–	–	–	–	–

Key: “Side” = the laterality of migraine attacks (B = Bilateral, L = Left unilateral, R = Right unilateral, U = Unilateral, alternating sides); “Pain w/o med” = average pain intensity reported during a migraine attack when no abortive medication is taken. Pain intensity was reported using a numeric rating scale from 0–10; “Pain w/med” = the average pain intensity reported during a migraine attack when abortive medication is taken. Freq = headache days per month.

Subjects verbally rated the pain intensity of their average migraine as a 5 or higher on a 0–10 scale, with 10 being the most intense pain imaginable. For those patients taking daily medications (e.g., preventive as opposed to acute medications to abort the attack), patients abstained from taking their migraine medications for one dosing interval prior to their scheduled scan session. Age- and gender-matched healthy subjects (8 females, 4 males; 31·7±7·2 years old) were also tested. Gender-matching was not exact, as the control group had one more male (and one less female) than the patient group.

### MR Acquisition

Imaging was conducted using a 3T Siemens Tim Trio scanner with a quadrature head coil. T_1_-weighted structural images were acquired using a 3D magnetization-prepared rapid gradient echo sequence (MPRAGE - 128 1.3 mm-thick slices with an in-plane resolution of 1 mm (256×256)). For functional resting state scans, a Gradient Echo (GE) echo planar imaging (EPI) sequence with TE/TR = 30/2000 was performed, with three hundred volumes captured for each scan. Each functional scan consisted of 34 slices oriented in an oblique plane to match the brainstem axis. Slices were 4.0 mm thick with an in-plane resolution of 3.5 mm (64×64). During these resting state scans, subjects were instructed to stay awake and to keep their eyes open.

### Image Analysis

Functional imaging datasets were processed and analyzed using scripts within FSL (FMRIB’s Software Library, www.fmrib.ox.ac.uk/fsl) [18]. The initial two volumes were removed from each of the functional scans to allow for signal equilibration. Visual screening of the functional volumes revealed that none of the subjects showed indications of gross movement (>1 voxel). The skull and other non-brain areas were extracted from the anatomical and functional scans using FSL’s script Brain Extraction Tool (BET). Motion Correction using FMRIB’s Linear Image Registration Tool (MCFLIRT) was performed on each functional scan. All volumes were mean-based intensity normalized by the same factor. The volumes were spatially smoothed with a 5 mm full-width at half-maximum (FWHM) filter, and a 150 s high-pass temporal filter was applied. These functional images were then co-registered with the anatomical images using FMRIB’s Linear Image Registration Tool (FLIRT), which uses an automated affine registration algorithm.

**Figure 1:**
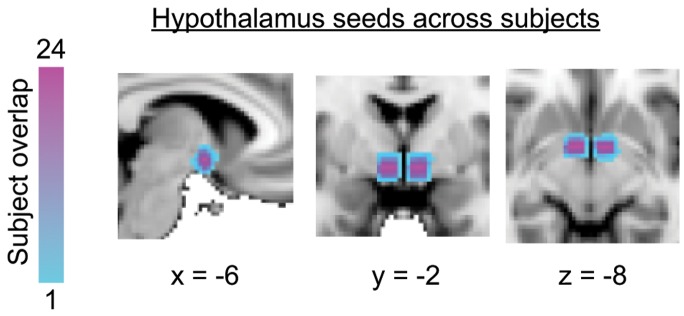
Hypothalamus seeds across subjects registered to the MNI152 standard brain. Anatomical boundaries for each subject were based on Saleem et al., 2007 (see Methods for details).

The hypothalamus was identified for each subject based on anatomical landmarks in the MPRAGE as described previously (**Figure 1**) [19]. A bilateral hypothalamic mask was conservatively defined for each subject using the following criteria: (1) the anterior extent was limited by the anterior commissure; (2) the inferior extent was limited by the mammillary bodies and optic tracts; (3) the posterior extent was limited by the mammillary bodies; (4) the medial extent was limited by the third ventricle; and (5) the region of interest extended 8 mm laterally from the medial extent.

First-level functional connectivity analysis of single subject data was performed using FMRI Expert Analysis Tool using FMRIB’s Improved Linear Model (FEAT FILM) Version 6.00 with local autocorrelation correction [20]. For single subject analysis, the mean time course for each corresponding hypothalamic mask was calculated, and entered as an explanatory variable (EV). Eight additional covariates of no interest were included that modeled motion (3 directions for rotation, 3 directions for translation), and the mean signal time courses measured in white matter and cerebrospinal fluid, as segmented by FMRIB’s Automated Segmentation Tool (FAST). The temporal derivative of the time course was not included as an explanatory variable. Subjects were spatially normalized to the MNI152 brain for group analysis.

Group functional connectivity maps were generated by fMRI expert analysis tool (FEAT) fMRIB’s Local Analysis of Mixed Effects (FLAME). A mixed effects contrast analysis was performed to compare migraine vs. control group functional connectivity. Statistical parametric maps were thresholded using Gaussian Mixture Modeling (GMM) [21], a multiple comparisons-based analysis which has previously been used in the context of detecting functional connectivity in brain imaging [22], [23]. A minimum cluster criterion of 7 voxels in original space (0.30 cm^3^) was implemented to identify significant clusters.

## Results

### Subjects

Twelve patients and twelve matched healthy controls were successfully scanned. All patients were had episodic migraine without aura. None were on preventive medications (**Table 1**). Medication use was either NSAIDs and/or triptans.

### Functional Measures

A mixed effects contrast analysis was performed to compare migraine vs. control group functional connectivity showed significant differences in a number of areas (details below).

### Hypothalamic Functional Connectivity

**Figure 2:**
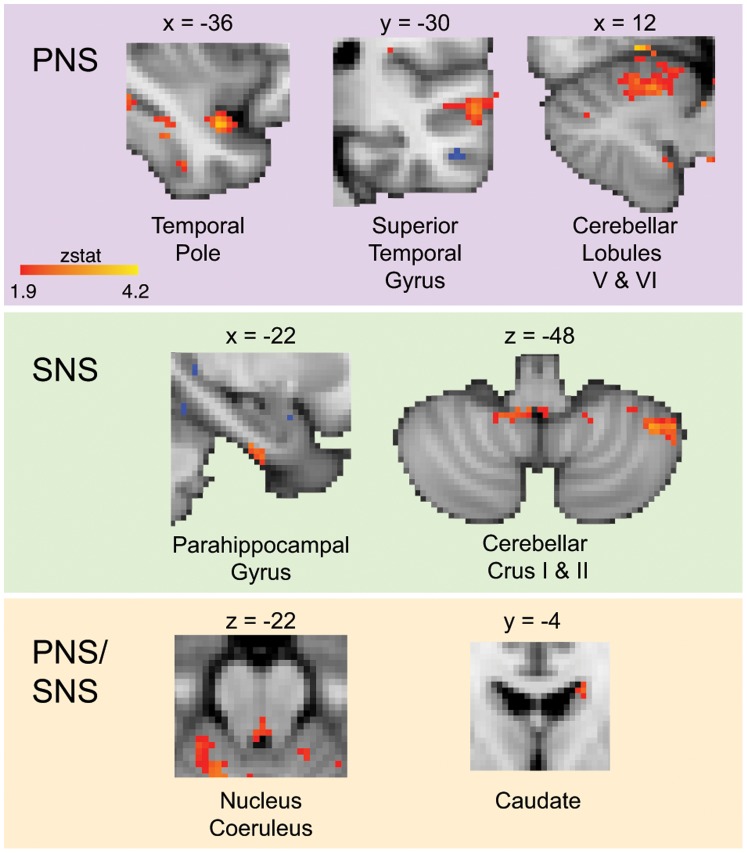
Increased hypothalamic functional connectivity in migraine-healthy controls in parasympathetic and sympathetic nervous system brain structures. Functional connectivity contrast maps were thresholded at a posterior probability of p>0.5 using GMM. Contrast maps overlay the standard MNI152 whole-brain atlas. PNS = parasympathetic nervous system, SNS = sympathetic nervous system. In reference to coordinates, x = sagittal (posterior-anterior, from left to right of the image), y = coronal (right-left), and z = axial planes (right-left).

Widespread differences in hypothalamic functional connectivity were detected in migraine patients vs. healthy control subjects. The majority of these differences occurred in brain regions related to sympathetic and/or parasympathetic nervous system processing, with migraine patients showing greater functional connectivity with these structures (**Figure 2** **;** **Table 2**). A neuroimaging meta-analysis of the central processing of autonomic function indicates that these regions can be categorized as sympathetic or parasympathetic nervous system structures [24], and are labeled accordingly in **Figure 2**.

**Table 2:** Brain regions with increased hypothalamic functional connectivity in migraine patients vs. healthy control subjects.

Brain Region	Lat.	z-stat	X	Y	Z	Vol (cm^3^)
**Frontal**						
PrCG	R	3.0019	6	−32	72	0.35
MFG	R	2.8577	44	22	44	0.41
**Parietal**						
SPL/SMG	L	2.8535	−46	−44	58	0.34
**Temporal**						
ITG	L	3.7500	−50	0	−38	0.31
Planum Polare	R	3.6963	46	−4	−16	0.66
TmP	L	3.6501	−36	6	−22	0.41
MTG	L	3.1737	−58	−10	−26	0.46
	L	2.9026	−58	−12	−14	0.73
PHG	L	3.1445	−22	−16	−28	0.42
	L	2.8751	−22	−12	−34	0.33
STG	L	3.1319	−62	−30	0	0.76
Hippocampus	R	3.0327	44	−16	−24	0.3
	L	2.9341	−48	−22	−22	0.46
**Sub-Cortical**						
Caudate	L	2.8124	−16	−4	24	0.5
**Brainstem/Cerebellum**						
Nucleus coeruleus	R	3.4440	8	−34	−26	0.58
PN	L	3.5626	−8	−24	−42	0.59
	R	2.9397	8	−20	−40	0.79
	R	3.0793	6	−26	−42	0.54
**Cerebellum**						
Cr I/II	L	3.3418	−40	−50	−48	0.85
V	L	3.2582	−12	−50	−14	0.78
	R	3.2184	14	−46	−16	0.52
	R	3.1642	12	−52	−20	1.19
	R	3.0691	6	−60	−22	0.86
	L	2.8326	−10	−60	−8	0.45
Verm VIIIa/VIIIb	L	3.2080	−2	−62	−36	0.33
Dentate nucleus	L	3.1411	−24	−50	−34	0.36
IX	R	3.0394	8	−46	−46	0.43
V/VI	R	2.8944	12	−58	−18	0.35

Legend: Cr I/II = Crus I and II; ITG = inferior temporal gyrus; IX = hemispheric lobule IX; MFG = middle frontal gyrus; PHG = parahippocampal gyrus; PN = pontine nuclei; PrCG = precentral gyrus; SMG = supramarginal gyrus; SPL = superior parietal lobule; STG = superior temporal gyrus; TmP = temporal pole; V = hemispheric lobule V; V/VI = hemispheric lobules V and VI; Verm VIIIa/VIIIb = Vermal lobules VIIIa/VIIIb.

Migraineurs showed enhanced functional connectivity with the hypothalamus in subcortical structures and throughout the temporal lobe (**Figure 2**). Areas of note included the nucleus coeruleus (volume: 0.58 cc, z-statistic: 3.44) pontine nuclei (volume: 1.92 cc, z-statistic: 9.58), caudate (volume: 0.50 cc, z-statistic: 2.81), cerebellar Crus I and II (volume: 0.85 cc, z-statistic: 3.34), temporal pole (volume: 0.41 cc, z-statistic: 3.65), superior temporal gyrus (volume: 0.76 cc, z-statistic: 3.13), hippocampus (volume: 0.76 cc, z-statistic: 5.97), and parahippocampal gyrus (volume: 0.75 cc, z-statistic: 6.02) (**Table 2**). Of these, the locus coeruleus and other pontine nuclei are of particular interest because of their role in stress and in the case of the locus coeruleus, as a major source of norepinephrine and has an excitatory effect on numerous brain regions including subcortical (e.g., amygdala, thalamus) and cortical structures [25], in addition to the hypothalamus and thus involved in autonomic function [26]. We interpret increased functional connectivity as enhanced or sensitized interactions between the hypothalamus and these structures. Conversely, migraineurs showed decreased functional connectivity localized to frontal and occipital lobe structures. These areas included the precentral gyrus (volume: 1.76 cc, z-statistic: 7.81), frontal pole (volume: 1.43 cc, z-statistic: 11.13), paracingulate gyrus (volume: 0.34 cc, z-statistic: 3.25), superior frontal gyrus (volume: 0.32 cc, z-statistic: 2.95), fusiform gyrus (volume: 0.46 cc, z-statistic: 4.20), and lingual gyrus (volume: 0.74 cc, z-statistic: 7.04) (**Table 3**). Here, decreased connectivity is interpreted and lower or diminished interactions or neural communications between the hypothalamus and structures noted.

**Table 3:** Brain regions with decreased hypothalamic functional connectivity in migraine patients vs. healthy control subjects.

Brain Region	Lat.	z-stat	X	Y	Z	Vol (cm^3^)
Frontal						
PrCG	R	3.9425	48	4	34	1.34
	R	3.872	24	−10	60	0.42
FrPole	L	3.8249	−40	38	16	0.41
	L	3.6729	−34	44	20	0.58
	L	3.6329	−28	38	28	0.44
ParaCG	L	3.2542	−4	40	32	0.34
SFG	R	2.9452	18	−2	64	0.32
Occipital						
Fusiform G	R	4.2019	16	−78	−12	0.46
Lingual G	L	3.7044	−4	−84	0	0.43
	L	3.3314	4	−86	−8	0.31

Legend: FrPole = frontal pole; ParaCG = paracingulate gyrus; PrCG = precentral gyrus; SFG = superior frontal gyrus.

## Discussion

This study found that interictal migraineurs have enhanced functional connectivity (FC) between the hypothalamus and brain structures related to autonomic function. Enhanced connectivity was observed to overlap with central representations of autonomic nervous system function, which has recently been characterized in a neuroimaging meta-analysis [24]. Our findings imply that these autonomic connections are sensitized in migraine patients, perhaps leading to increased autonomic symptoms associated with ictal events in migraine. As discussed below, anatomical correlates between the hypothalamus and the regions noted are present.

### Hypothalamo-Sympathetic FC

In migraineurs, the hypothalamus demonstrated increased functional connectivity with sympathetic nervous system structures, such as the parahippocampal gyrus and cerebellar Crus I and II. The hypothalamus is structurally connected to the hippocampus through the fornix [27], and to the cerebellum through hypothalamo-cerebellar connections [28]. Enhanced connectivity with these sympathetic structures may prime cortical responses to external stressors relating to anxiety, memory, spatial location, and aversive stimuli [29]–[31]. BOLD signals in these hippocampal and cerebellar regions have also previously been found to co-vary with sympathetic activity in the form of skin blood flow measures and skin conductance response [32]. In this previous experiment, sympathetic responses were elicited using an aversive conditioning paradigm and measured during anticipation of noxious heat and the painful experience itself. While the authors acknowledged a potential confound between sympathetic responses with motor tasks and sensory stimuli in their data, we were able to see a hypothalamic link with these structures at rest without confounds stemming from explicit motor tasks or sensory stimuli.

### Hypothalamo-Parasympathetic FC

Migraineurs also showed increased hypothalamic connectivity with parasympathetic nervous system structures, including the temporal pole, superior temporal gyrus, and cerebellar lobules V and VI. Structural connectivity between these areas and the hypothalamus has been established previously [28], [33]. We have shown recently that the temporal pole is hyperexcitable in migraine patients [22]. This structure, along with the superior temporal pole, may be involved in the interictal hypersensitivity to smell and light [34], [35]. Cerebellar lobules V and VI have been related to a wide variety of tasks, including cognitive and emotional processing [31], [36]. The enhanced functional connectivity between the hypothalamus and these parasympathetic brain regions allows them to interact in ways that may impact interoceptive processes in migraine patients.

### Locus Coeruleus and Caudate Nucleus

Structures related to both sympathetic and parasympathetic processing, such as the locus coeruleus (LC), also exhibited increased hypothalamic connectivity. The LC is the largest noradrenergic nucleus in the brain. Through heavy innervation of multiple forebrain regions including the hypothalamus [25], [26], it is involved in number of vital functions including wakefulness [37], responses to stress [38], and regulation of emotion [39]. Although a specific role in migraine is unknown, LC involvement in the inhibition of nociceptive reflexes [40] and firing mode of thalamic and prefrontal cortex neurons in response to noxious stimuli [41] raise the possibility that it may also be involved in normal (and perhaps abnormal) pain modulation during migraine.

Another structure showing altered hypothalamic FC that is implicated in sympathetic and parasympathetic function is the caudate nucleus. Efferent connections between the hypothalamus and the caudate have been shown in tracing studies in the rat [42]. The caudate has also recently been associated with arousal [43], but is also related to motivation, learning and memory, and pain and sensory processing [44], [45]. Increased hypothalamic connectivity with these structures may be responsible for the recurring chronobiological features of migraine [1], [2] and appetitive drive [46].

The data indicates decreased functional connectivity with a number of brain regions of migraineurs vs. healthy controls (**Table 3**). These include cortical regions in the frontal and occipital regions. Hypothalamic connections to the frontal lobes have been documented in monkeys [47]. While unknown, the decreased FC between frontal regions may be specific to diminished defined functions. For example, these may be anti-correlated (potentially related to parasympathetic processes) to sympathetic hypothalamic drive [24], [48]. Furthermore, regions such as the fusiform gyrus, also showing diminished decreased functional connectivity with the hypothalamus may relate to autonomic responses to emotional stimuli [49].

The study did not differentiate between migraine patients with and without aura. Migraine with aura is a more aggressive disease, at least based on observed brain changes [50]. Based on alterations in autonomic function in patients reported with aura [51], [52] we would expect that such patients would have functional connectivity further diminished when compared with migraineurs without aura.

## Conclusions

While the resting state connectivity data suggests that the hypothalamus has widespread influence on autonomic nervous system structures in migraine patients, it does not necessarily indicate that the hypothalamus has a central role in generating migraines. The connectivity results are correlative, and the inference of functional impact is based on previous studies. However, the results do indicate that changes in hypothalamic connectivity are a central feature in migraine patients, and may be responsible for the manifestation of autonomic symptoms. While other autonomic brain systems must clearly play a role in migraine, only those areas noted in the results showed differences between healthy subjects and controls for the resting state data acquired. Thus, measures of hypothalamic hyperactivity to a stressor (e.g., heat or a migraine attack) or measures of hypothalamic hormones would contribute to our understanding of the structure in the migraine condition.
